# Soil biological activity after a sixty-year fertilization practice in a wheat-maize crop rotation

**DOI:** 10.1371/journal.pone.0292125

**Published:** 2023-09-28

**Authors:** Anna Füzy, István Parádi, Bettina Kelemen, Ramóna Kovács, Imre Cseresnyés, Tibor Szili-Kovács, Tamás Árendás, Nándor Fodor, Tünde Takács

**Affiliations:** 1 Department of Soil Biology, Institute for Soil Sciences, Centre for Agricultural Research, Budapest, Hungary; 2 Department of Plant Physiology and Molecular Plant Biology, Eötvös Loránd University, Budapest, Hungary; 3 Department of Soil Physics and Water Management, Institute for Soil Sciences, Centre for Agricultural Research, Budapest, Hungary; 4 Crop Production Department, Agricultural Institute, Centre for Agricultural Research, Martonvásár, Hungary; Universidade Federal de Minas Gerais, BRAZIL

## Abstract

This study aimed to survey the long-term effects of fertilization practices on the functional diversity of the soil microbiota. A 60-year fertilization experiment with mineral fertilizers, farmyard manure and combined treatments was sampled in two consecutive years in maize (*Zea mays* L.) and wheat (*Triticum aestivum* L.). Soil chemical properties, plant growth and physiological parameters were measured. The MicroResp^TM^ method was applied to assess the community level physiological profiles (CLPPs) of the rhizosphere soil, and the arbuscular mycorrhizal fungal (AMF) colonization of the roots was determined. Samples were taken in the early vegetative stages, at flowering, and at harvest in both years. The measured parameters were analysed using multifactorial ANOVA to determine treatment effects, crop-dependent differences, and seasonality. PCA analysis was performed on the data matrix to reveal more complex correspondences, and Pearson’s product-moment correlation was used to confirm relationships between some of the measured soil and plant parameters. Fertilization treatments caused long-term changes in some biological parameters such as: MicroResp^TM^ parameters, citrate utilization, total substrate-induced respiration value, and the ratio of utilization of amino acids and sugars. The rate of AMF colonization responded mainly to the plant nutrition status and the plant requirements, suggesting a plant-mediated effect in the case of mycorrhiza. Mineral nitrogen fertilization and soil acidification were found to be the main factors affecting the catabolic activity of soil microbiota, while AMF colonization responded to the balance of plant nutrition.

## Introduction

The main aim of annual fertilization and regular manuring is to establish long-term yield stability. Continous field experiments have provided a huge amount of data on yield stability as well as on many of other parameters, which are well discussed in the results published from the long-term experiment studies in the present paper [1–3] and from other similar ones [4–7]. An increase in macronutrients may contribute to higher yield, depending on the crop species, previous cropping regime [2], meteorological conditions, or the balance of fertilization. Soil macronutrients are important for yield stability, though they are not the only factor affecting sustainability. Unbalanced nutrition, including micronutrients, changes the soil properties, such as pH or aggregate formation, and causes shifts in soil microbiota ‐ the invisible part of soil health. These could reduce or eliminate the effect of fertilizers, making the system performance unsustainable [8–10]. The natural resilience of soils is crucial for sustainable agriculture [11].

Investigations into the biological part of soil fertility and health has only started recently, and the methodology used is very diverse. Community level investigations on the soil microbiota provide information only on a very small part of the whole system, but can provide useful data about the degree and direction of shifts in the microbiota. Several methods are used to assess the composition, diversity or functioning of soil microbial communities. RNA- or DNA-based methods like DGGE (Denaturing gradient gel electrophoresis) [8,12] community sequencing [13–16], substrate utilization profile assays [17], enzyme activity measurements [12] and fatty acid methyl ester analysis fingerprinting [9,18] are only some of the currently applied methods [19].

The MicroResp^TM^ technique is commonly used to assess the community level physiological profiles (CLPP) to monitor soil health and quality [20]. In this method, the whole soil sample is incubated and analysed, thus establishing a complete picture of the catabolic ability of the soil microbiota through substrate induced respiration (SIR) measurements and the functional diversity of the soil [21]. The utilization pattern of C-source substrates (e.g. sugars, amino acids, organic acids) may indicate differences in the microbial community composition of soils.

Arbuscular mycorrhizal fungi (AMF) are widespread obligate root microsymbionts with low host specificity [22,23]. The functional and genetic diversity of indigenous soil AMF can be good indicators of soil health in agricultural systems with different crops or crop rotations. The AMF colonization rate may be influenced by the soil (even if it’s with the mediation of the plant partner) and the host plant [24].

In most cases phosphorus and nitrogen are two essential macronutrients that regulate plant growth. Both the plant-associated bacteria and the symbiotic or free-living fungi may have a crucial role in controlling nutrient mobilization, immobilization, and element transport processes in the soil [25]. In a cooperative way with the AMF, the bacterial communities in the mycorrhizosphere and hyphosphere promote the P solubilisation and N fertilization. Soil nutrient levels influence AMF colonization activity, thus affecting the coexistence of plant species and microbes [26]. Soil P supply may change the symbiotic cost-to-benefit ratio [27]. P limitation increases the presence of AM fungal structures in the root system, whereas a high concentration of available phosphorus decreases successful AMF colonization rates.

Apart from phosphates, external AM fungal hyphae can absorb NO_3_^-^ and NH_4_^+^, utilize organic N sources and possibly activate N transporters in the plant. Over-fertilization of N decreases the number of appressoria (the entry points of AMF) and the intensity of AMF root colonization. However, organic matter addition to the soil may increase the P available for plants, leading to a lower mycorrhizal dependence. The soil N/P and C/P element ratios may regulate the microbial community composition and enzyme activities [28,29].

A 60-year NPK fertilization field experiment provided the framework for the present investigations, where the following questions were addressed: 1. What are the main effects of long-term soil fertilization practice on the soil physicochemical and biological parameters? 2. How the crop plant modifies the soil biological parameters? 3. Which soil and plant parameters can indicate the higher expected yield?

Two “spatial” factors (chemical fertilizer applications and farmyard manure addition) and two temporal factors (seasonal–three growth stages of plants, interannual ‐ 2017 and 2018) were used in the factorial designed experiment to select the strongest aspects affecting physicochemical parameters, and the microbiological changes in soil.

## Material and methods

### Experimental design

**Fig 1:**
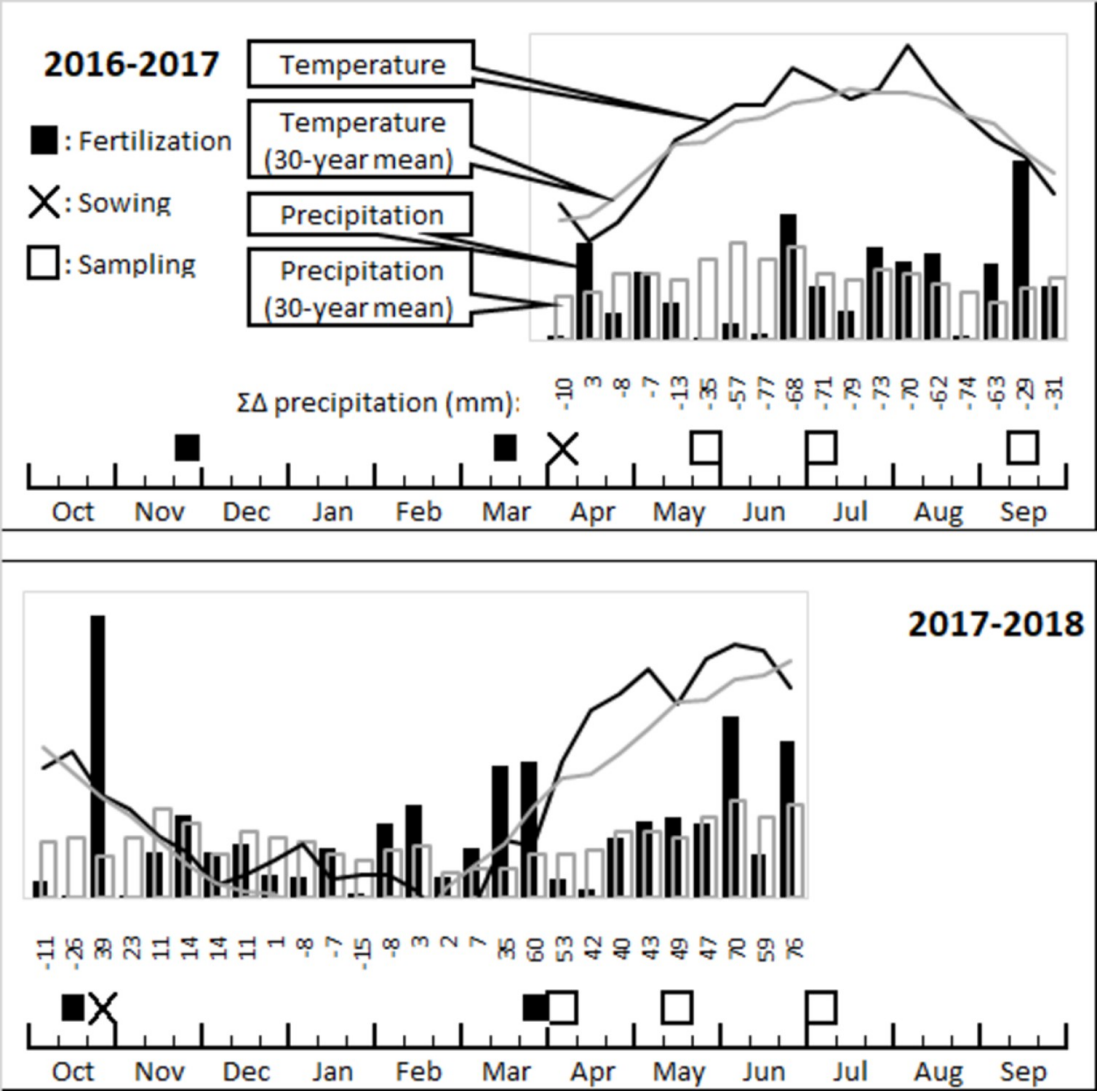
Time schedule of the experiment. Fertilization, sowing and sampling in 2017–18, temperature and rainfall data for the growing season.

The long-term fertilization experiment was set up in 1959 at Martonvásár, Hungary (N 47°18’41”, E 18°46’50”), in the experimental station of the Centre for Agricultural Research. No permission was necessary from other authorities for sampling and monitoring. According to the FAO-WRB classification system [30], the soil is a Haplic Chernozem, with 51.4% sand, 34.0% silt and 14.6% clay; bulk density 1.47 g cm^-3^, pH(H_2_O) 7.3, 0–1% CaCO_3_ content, and 3.2% soil organic matter content. Based on the recommendations of the Hungarian plant nutrition advisory system [31], the plant-available macronutrient supply in the soil was poor for P, and medium to good for K. The climate of the area is continental with a 30-year average temperature of 11.0°C (–1°C in January and 21.2°C in July) and an annual rainfall of 548 mm, based on data from the on-site weather station. The treatments were arranged in a random block design with 6 × 8 m plots. Eight different treatments were tested: Control, only N, only P, NPK mineral fertilizers–with farmyard manure; Control, only N, only P, NPK–without farmyard manure. These treatments were applied from 1959. Fertilizer doses of 160 kg ha^-1^ N, 80 kg ha^-1^ P, 80 kg ha^-1^ K were added to the soil from 1976 onwards. Previously, slightly different doses were applied from 1959 to 1975. All treatments were set up in three replicates. The crops in the four-year fertilizer cycles were maize in the 1^st^ and 2^nd^ year, winter wheat in the 3^rd^ and 4^th^ year. Half the N doses (NH_4_NO_3_) and all P (P_2_O_5_) and K (K_2_O) fertilizers were distributed before soil cultivation in October or November. The remaining N were added before sowing or as topdressing in early spring. Farmyard manure was applied once every four years at a rate of 40 t ha^-1^ in autumn, for the last time in 2015, before the sampling. The experiment timelines for 2017 (mid-season grain hybrid maize–*Zea mays* L. cv. Mv Tarján, FAO 380) and 2018 (winter wheat, early maturity group–*Triticum aestivum* L. cv. Mv Nemere, 2014 CPVO 42131) are illustrated in Fig 1.

### Sampling and monitoring

We performed three sampling per year in 2017 and 2018, at leaf development and stem elongation stage (BBCH: 14–16 for maize, 31–33 for wheat; [32]), at flowering (BBCH: 61–65 for maize, 61–62 for wheat), and at full maturity (BBCH: 89) (Fig 1). We collected pooled samples of three aboveground plant biomass from each plot. Approximately 1 kg of soil samples were taken from the rhizosphere (0–20 cm depth) and air-dried for chemical analysis or stored at 4°C until used in MicroResp^TM^ measurements. Root samples were collected, and approximately 1 g fresh weight was stored in 70% ethanol. Plant growth was monitored by stem diameter measurement for maize and complete shoot dry mass for wheat at sampling times. We determined the grain yield at harvest. Chlorophyll content (SPAD value) was measured on the youngest fully developed leaf with a SPAD-502 meter (Konica Minolta Inc., Osaka, Japan) at the early growth stage (4–5 leaf stage) and at flowering.

### Soil chemical analysis

Soil chemical parameters were measured from rhizosphere soil samples, that were air-dried and sieved (mesh size <2 mm) before analysis. Measurements were carried out according to the methodology of the Hungarian standards: soil organic carbon as soil humus [33], soil pH [34], plant available P and K concentrations with ammonium-acetate lactate extraction [35,36], total N content of the soil [37], and soil NH_4_-N and NO_3_-N concentrations from KCl extracts [35]. We determined soil Ca concentrations in AL-extract using an inductively coupled plasma atomic emission spectroscopy (ICP-AES, Jobin-Yvon Ultima 2 sequential instrument).

### Assessment of community level physiological profiles

The MicroResp^TM^ method was applied to assess community level physiological profiles (CLPPs) [38], using the protocol provided by the manufacturer (The James Hutton Institute, Craigiebuckler, Aberdeen, UK). We sieved the rhizosphere soil (mesh size <2 mm) and wetted with sterile distilled water it to 50% of water-holding capacity. From each sample, about 40 g soil were filled into deep well plates, covered with Parafilm M, and incubated in a desiccator for 5 days in dark and at room temperature. 23 different carbon sources were used for the measurements (see Table 1), in 4 repetitions on each plate, and distilled water as control; the pH of the substrates was adjusted to 7.0. The plates were sealed with detector plates containing cresol-red indicator and incubated at 25°C. Substrate utilization patterns were measured at 570 nm with a microplate reader (Anthos 2010, Biochrom, Cambridge, UK) twice: just before closing the plate and 5 h later. The change in absorbance was converted into % CO_2_ values using the equation given by the manufacturer. The detector plates were calibrated prior to the experiment at different CO_2_ concentrations using a gas chromatograph (Fisons GC 8000, Rodano, Italy). The functional evenness index was also calculated for the 23 substrates according to Magurran [39].

**Table 1:** Applied substrates at Microresp^TM^ method. The name, abbreviation and concentration of 23 different substrates.

Substrate	abbr.	cc. (g l^-1^)
***Sugars***		
D-glucose	GLC	32.0
D-fructose	FRU	32.0
D-(+)-galactose	GAL	32.0
D-mannose	MAN	32.0
L-(+)-arabinose	ARA	32.0
D-xylose	XYL	32.0
Trehalose	TRE	33.7
L-rhamnose	RHA	35.1
***Polyols***		
Myo-Inositol	INO	32.0
D-mannitol	MAT	31.6
D-sorbitol	SOR	31.6
***Carboxylic acids***		
Citric acid-monohydrate	CIT	13.7
DL-malic acid	MAL	14.3
Na-succinate	SUC	11.9
Gluconic-acid-potassium	GLA	12.3
3,4 dihydroxybenzoic acid	DHB	6.5
***Amino acids***		
L-asparagine-monohydrate	ASN	6.4
L-glutamine	GLN	8.2
L-serine	SER	13.7
L-glutamic acid	GLU	4.9
L-lysine hydrochloride	LYS	15.8
L-arginine	ARG	5.0
L-alanine	ALA	16.2

### Arbuscular mycorrhizal colonization of roots

The arbuscular mycorrhizal fungal colonization of the roots was determined after clearing and staining the root samples [40]. After microscopic observation (BX51 Olympus, Tokyo, Japan), the fungal colonization intensity (M%) and the arbuscule richness of the roots (A%) were calculated according to the five-class method described by Trouvelot et al. [41].

### Statistical analysis

The statistical analysis was performed using R-statistic software [42]. Factorial ANOVA was carried out for all the measured parameters, 4-way ANOVA for soil parameters and substrate-induced respiration (SIR) values, and 2-way analysis for plant data and root microsymbiont parameters. The following factors were used: year (F1) and season (F2) of sampling, organic manure (F3) and mineral fertilization (F4) treatments. The reduction of factors to F3 and F4 is reasonable for plant and plant-associated parameters, as there are annual differences for the plant species and plant growth stages. The statistical analysis was performed separately for each sampling time for F3 and F4. If the prerequisites of variance analysis were violated, an aligned rank transformation (ART) was made [43]. Significant differences between mean values were determined using the Tukey or Nemenyi post-hoc test.

Pearson’s product-moment correlation was calculated between the data series. PCA (principal component analysis) was used to analyse the data matrix of the soil samples and SIR values. SIR values were square root transformed before statistical analysis, and PCA analysis for the 23 substrates and correlation analysis with citrate were performed on a normalized data set (values divided by the average of all substrates [38]).

## Results

### Temporal factors in the experiment (F1 and F2)

**Fig 2:**
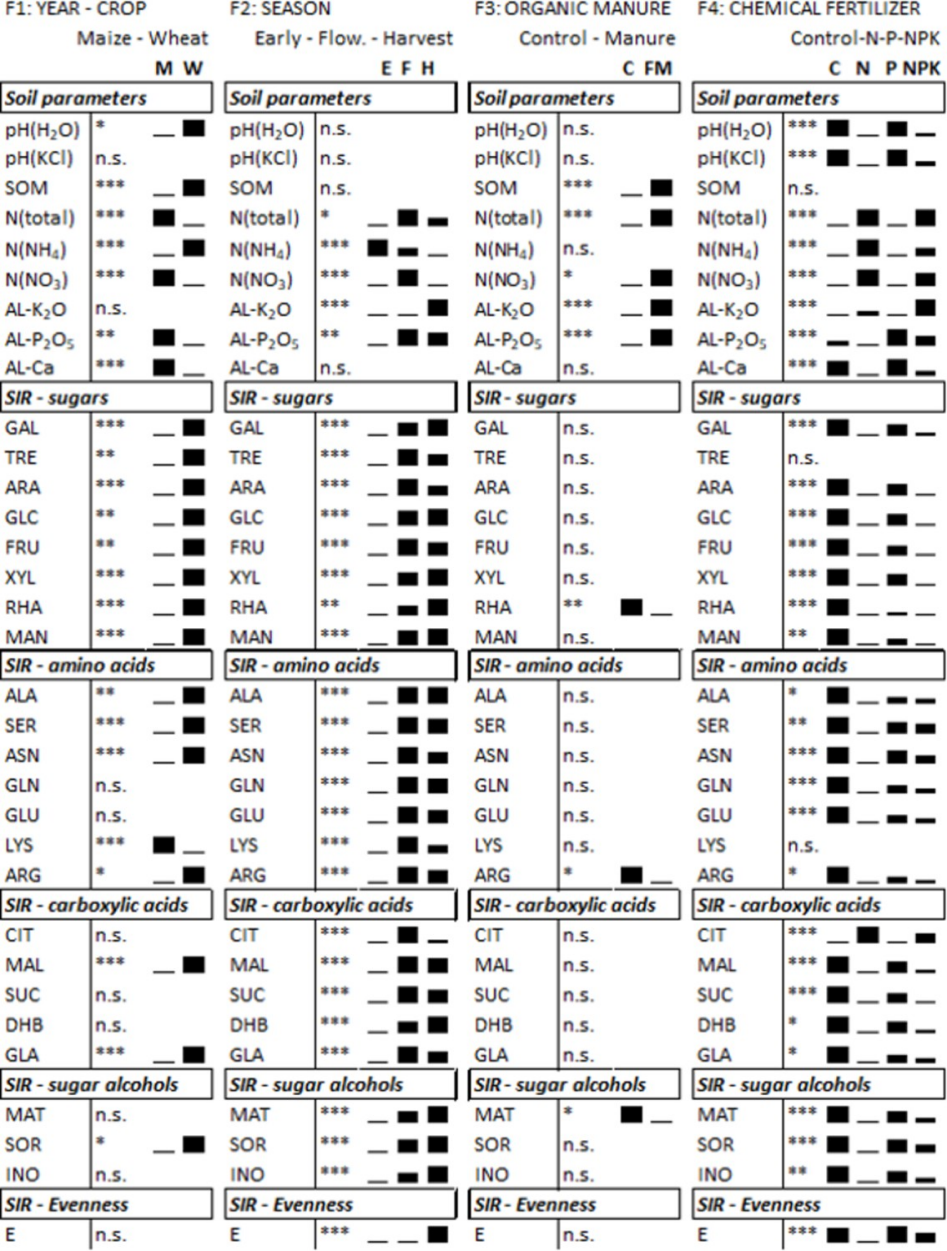
Results of 4-way ANOVA on soil chemical parameters and substrate-induced respiration (SIR) values. The table presents significance levels and the bars reveal the relationships between mean values. Factor 1 (F1): Comparison of the 2 years. Factor 2 (F2): Comparison of sampling times in the growing season. Factor 3 (F3): Comparison of treatments with and without farmyard manure. Factor 4 (F4): Comparison of mineral fertilizers treatments. Abbreviatons of SIR substrates are the same as in Table 1. The figures with the measured data and detailed ANOVA-tables are in supplement. n.s.: non-significant, *: p<0.05, **: p<0.01, ***: p<0.001. M: Maize, W: Wheat, E: Early sampling, F: Flowering, H: Harvest, FM: Farmyard manure.

In the case of the year effect (F1), we found several significant differences between the soil macronutrient concentrations (Fig 2). Differences in the concentration of N forms (NO_3_^-^ or NH_4_^+^) probably caused by the different sampling times. Although maize and wheat were sampled at the same growth stage, the time interval between fertilization and sampling differed for the two crops (Fig 1). The microbial activity, characterized by SIR, was more pronounced for the sugars and some amino acids in wheat samples, and lower only in the case of lysine.

We observed clear seasonal dynamics (F2) for the levels of N and P forms in the soil and for the SIR values. The most intensive respiration was detected at the flowering stage (F), which had values much higher than those of the young plants (E) (Fig 2).

### Long term fertilization treatments as factors (F3 and F4)

**Fig 3:**
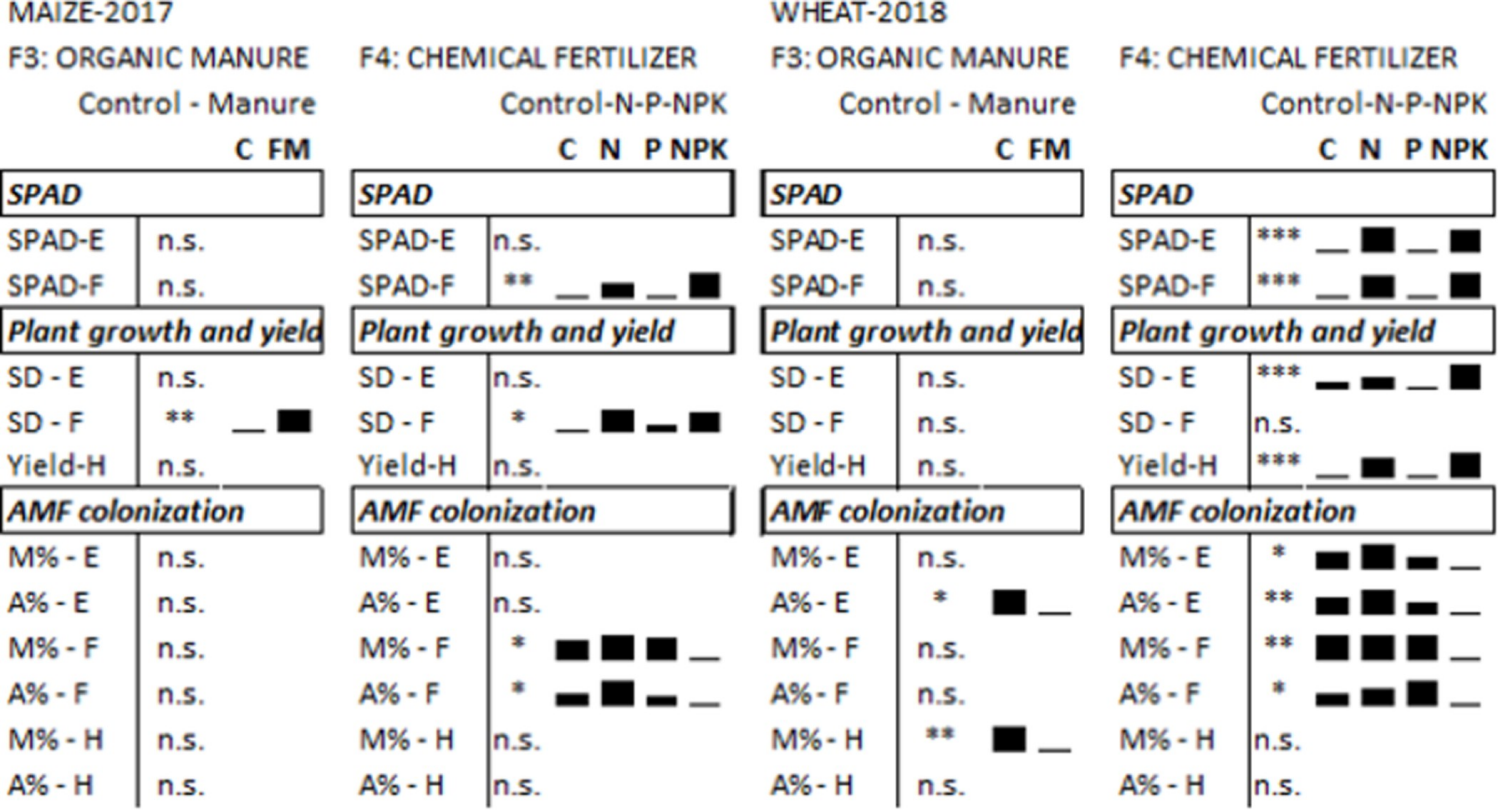
Results of 2-way ANOVA on plant growth, yield and AMF root colonization rate. The table presents significance levels and the bars reveal the relationships between mean values. Factor 3 (F3): Comparison of treatments with and without farmyard manure. Factor 4 (F4): Comparison of mineral fertilizers treatments. The figures with the measured data and detailed ANOVA-tables are in supplement. n.s.: non-significant, *: p<0.05, **: p<0.01, ***: p<0.001. SD: Shoot diameter, SDM: Shoot dry mass. E: Early sampling, F: Flowering, H: Harvest, FM: Farmyard manure.

Farmyard manure (FM) treatments (F3) resulted in the lowest number of significant differences: only the soil organic matter and macronutrients values were increased (total-N, NO_3_^—^N, AL-K_2_O, AL-P_2_O_5_; Fig 2). These changes did not affect the soil respiration pattern, except for the rhamnose-induced respiration, which was significantly higher in FM-treated plots (Fig 2). Manure treatment resulted only a few significant differences in plant growth, physiology, and symbiotic relationships. In the case of wheat, colonization parameters, arbuscularity in the early plant phenophase [A% (E)] and mycorrhization intensity at harvest [M% (H)] were significantly higher in the control than in manure-treated plots (Fig 3).

The application of chemical fertilizers (F4) had a strongly affected soil chemical properties and root-associated microbial activities. Chemical fertilizers resulted in acidification of the originally slightly alkaline soil. All chemical soil parameters changed in the treated plots, except for the soil organic matter content. The SIR rates of rhizosphere microorganisms typically decreased in the N- and NPK-treated plots, whereas their citrate-induced respiration was higher. In the case of wheat in 2018, N fertilizers improved plant growth and crop yield more effectively. The complex fertilizer, NPK, caused a slight decrease in the AMF colonization of plant roots (Figs 2 and 3).

### Soil chemical parameters and substrate-induced respiration

**Fig 4:**
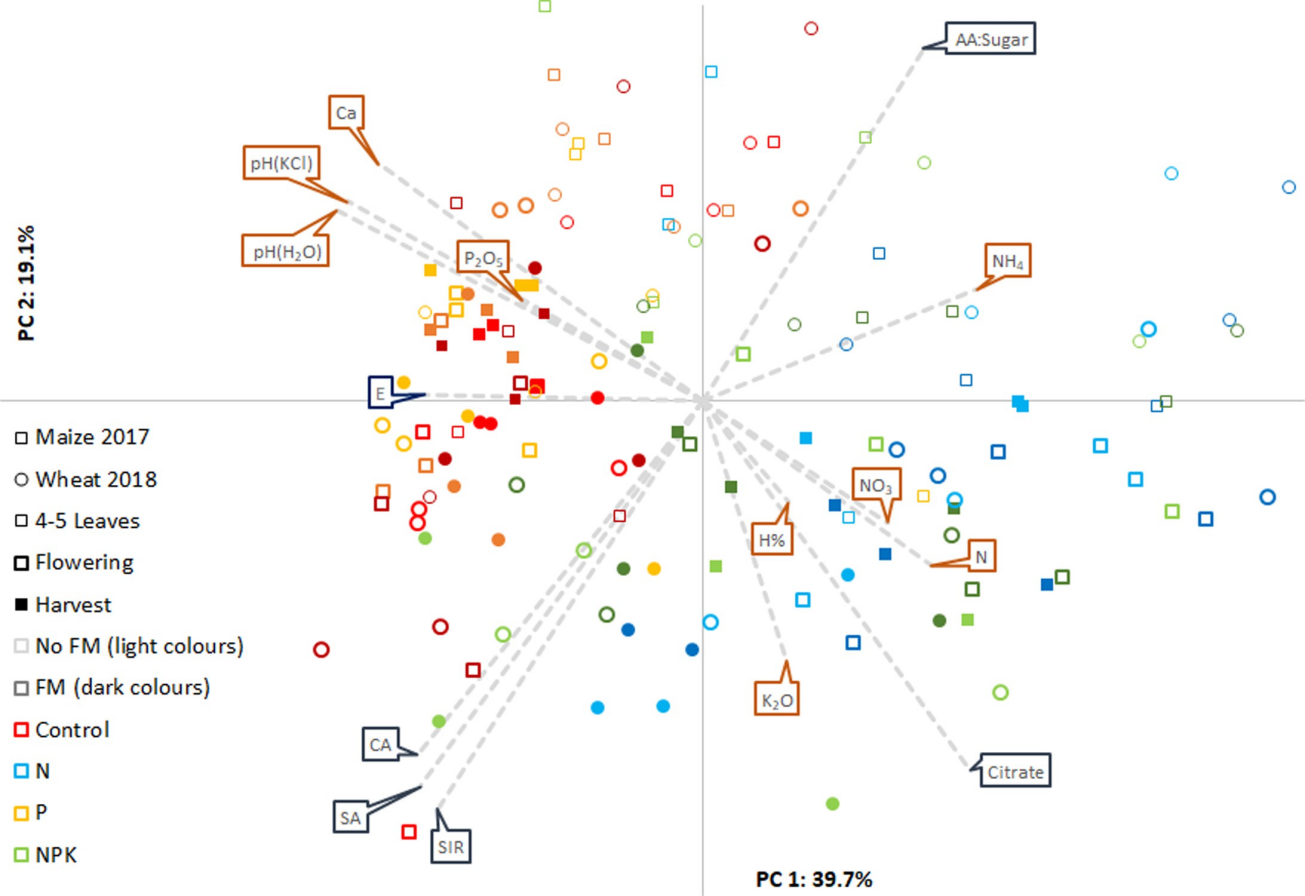
Scaled PCA biplot of chemical soil parameters (brown text boxes) and SIR values (blue text boxes). Each sample was marked separately as points according to the treatments (colour of flags ‐ with farmyard manure (FM): Dark colours, without farmyard manure: Light colours) and sampling events (shape of flags for the year, borderline and filling of flags for sampled season), while the vectors were the measured parameters. The SIR values were grouped as carboxylic acids (CA), sugar alcohols (SA), and as the ratio of amino acids to sugars (AA:Sugar). SIR from citrate-utilization (Citrate) was not grouped to the others, and a calculated factor, functional evenness (E) was also presented.

Fig 4 illustrates the results of PCA analysis on soil chemical parameters and the SIR values of grouped or selected substrates. Preliminary analysis revealed the greatest differences between the samples for SIR values, like the utilization of citrate or the ratio of amino acids and sugars, so these were used in the complex analysis. Principal components 1 (PC1) and 2 (PC2) explained about 60% of the total data variance. PC1 was determined by some soil macronutrient (N-forms, P, Ca) concentrations, soil pH, the functional evenness factor and citrate-utilization and the sample points, as the objects of PCA analysis separated in the two-dimensional space according to N fertilization treatments. In contrast, PC2 was determined mainly by the SIR values, and was slightly modified by available nitrogen forms like NH_4_^+^ or NO_3_^-^. The samples were separated according to sampling time: early sampling resulted in lower SIR values, higher ammonium-N in the soil, and a high relative value of amino acid utilization. No separation could be seen between phosphate fertilization and the control treatments, nor between soil samples given farmyard manure and the control plots.

**Fig 5:**
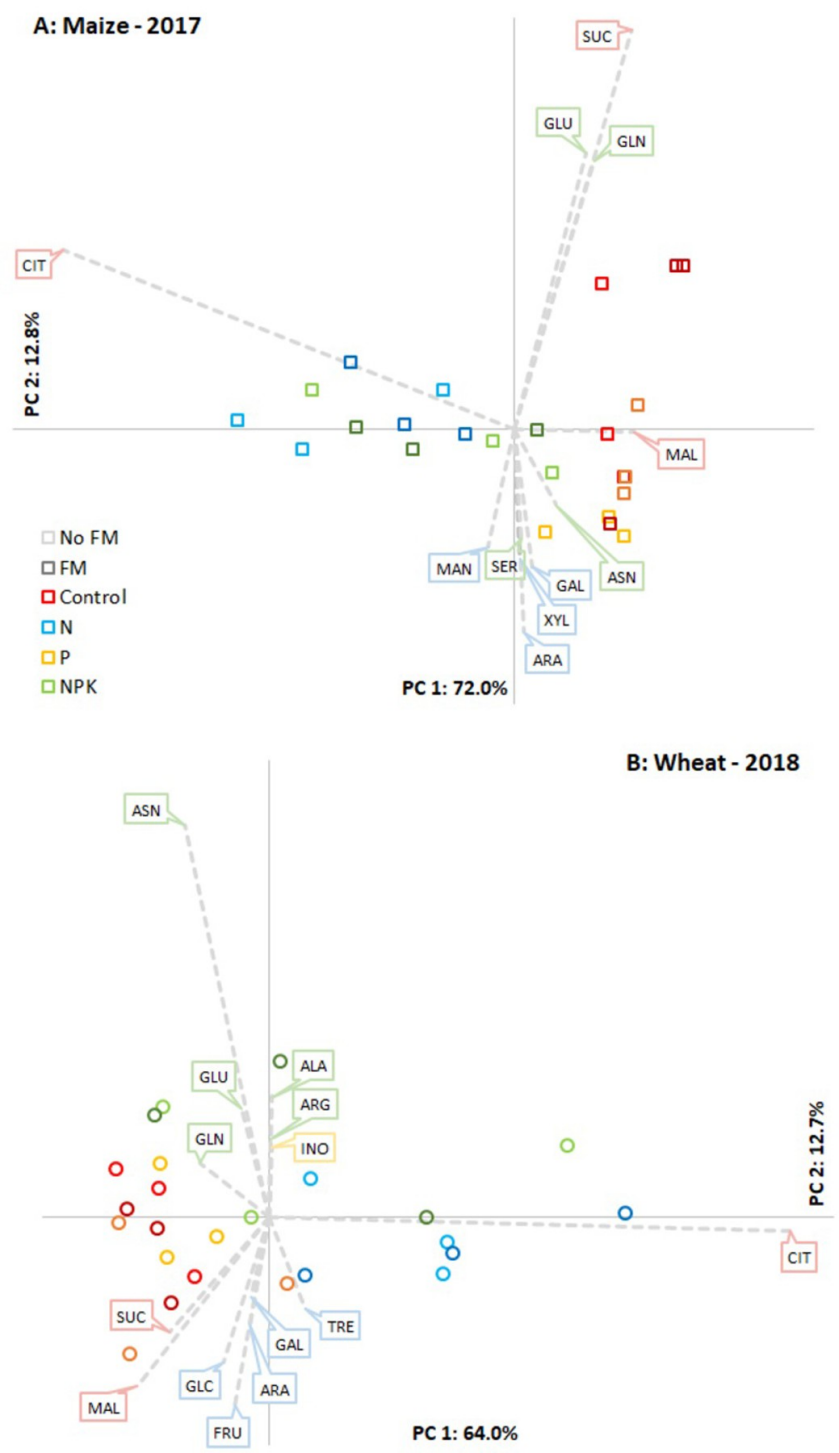
Unscaled PCA biplots for the relative SIR values of 23 different substrates at flowering for maize (A) and wheat (B). Substrates are presented on the biplots as vectors, if the PC1 or PC2 coordinate value was higher than 0.1, or less than -0.1.

The highest absolute SIR values were measured at the flowering stage (Fig 2), so PCA analysis with relative SIR values was carried out for this most active period of soil microbiota on the 23 different substrates (Fig 5). The citrate utilization rate was a particularly strong factor in the analysis. Principal component 1 contained mainly this vector and explained more than 60% of data variance for both crops. Sample points dispersed along PC1 according to N fertilization treatments: N and NPK treated plots moved together with the citrate-vector, while P and control treated plots moved in the opposite way. PC2 explained only 13% of the total variance, and the orientation of samples and vectors along this ordinate was not the same for the two crops in 2017 and 2018. Though not a clear separation, a certain orientation tendency was visible along PC2 between the control samples and P treatments in the first year for maize. The soil respiration induced by succinate, glutamine and glutamic acid was higher in the control treatment, whereas P-added soils responded more to sugar substrates. In the case of wheat, PC2 separated the N treatment from the complex NPK treatment. The main vector at NPK side was amino acid substrates, while the other direction was determined by sugar substrates.

### Citrate-induced respiration and soil chemistry

**Fig 6:**
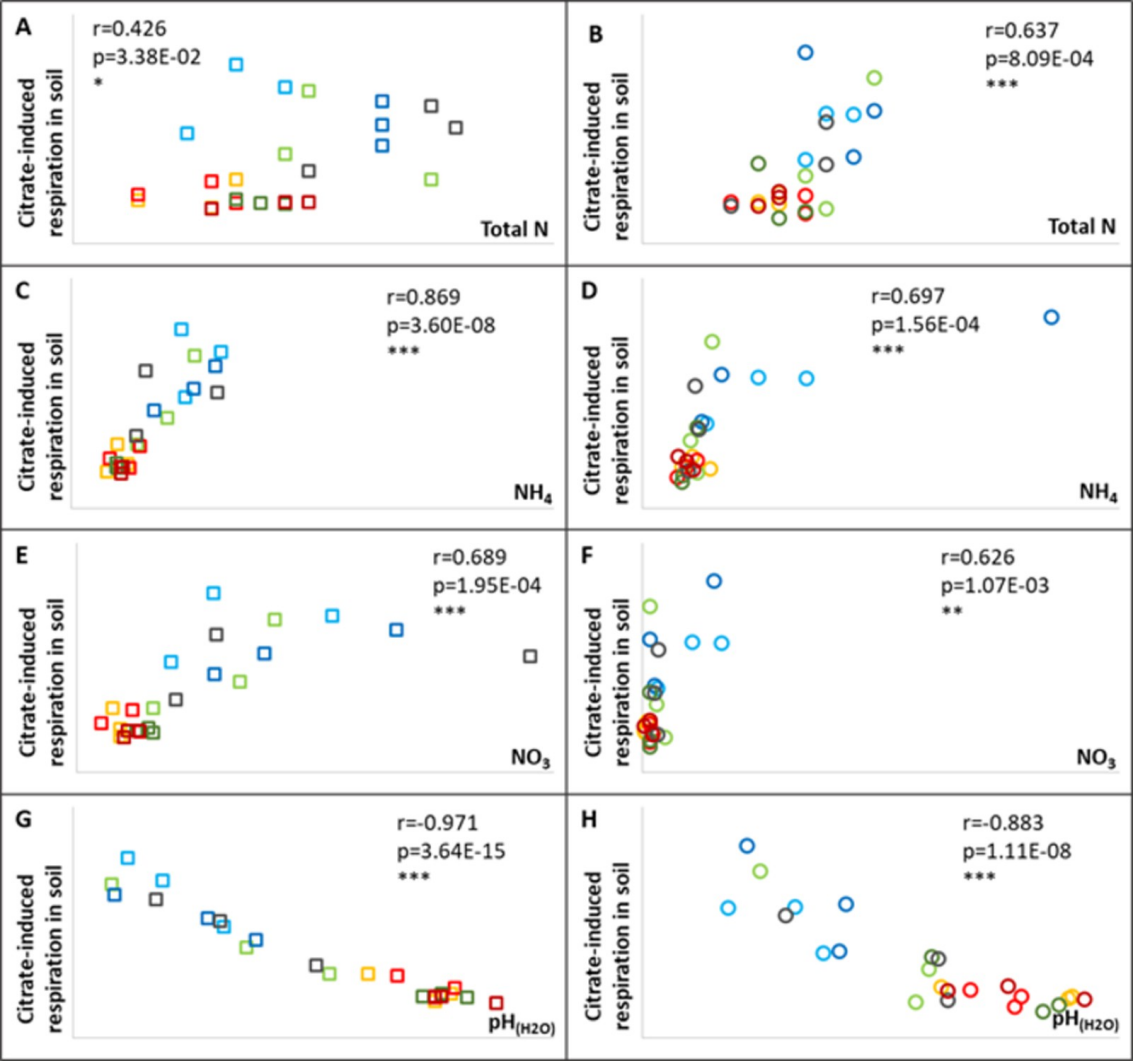
Pearson’s product-moment correlation analysis between citrate-induced respiration and N forms in the soil (A-F) and between citrate-induced respiration and soil pH (G-H) in the flowering stage of maize (A, C, E, G) and wheat (B, D, F, H). The key of the symbols is the same as at Fig 4. (Control–red, N–blue, P–yellow, NPK–green, light colours: Without FM manure, dark colours: With FM manure). n.s.: non-significant, *: p<0.05, **: p<0.01, ***: p<0.001.

Citrate-induced respiration was a particularly strong factor, acting as the main determinant of the variance between the samples (Fig 2), specifically between treatments with or without chemical N fertilizers. The Pearson’s product-moment correlation analysis revealed close negative relationship between citrate-induced soil respiration and several chemical soil parameters (Fig 6). The analysis was made using samples collected at the flowering stage: the most active period of soil microbiota. Significant correlations were found between the SIR values of citrate and all the N forms in the soil for both crops (Fig 6A–6F). The strongest correlation was revealed between citrate SIR and soil NH_4_-N, and the weakest with total N for the maize crop in 2017. The Pearson’s product-moment correlation analysis showed an extremely strong relationship (Fig 6G and 6H) between citrate utilization and soil pH, especially for maize (R^2^ = 0.943).

### Plant growth, plant physiology, AMF colonization parameters and yield stability

We found a fertilization effect on grain yield for winter wheat in 2018: chemical fertilizers containing nitrogen (N, NPK) significantly increased the crop yield (Fig 3). The N, NPK, and the organic manure treatments increased the plant growth parameters and SPAD values of both crops at the flowering stage, but no effect was manifested on the yield of maize.

**Fig 7:**
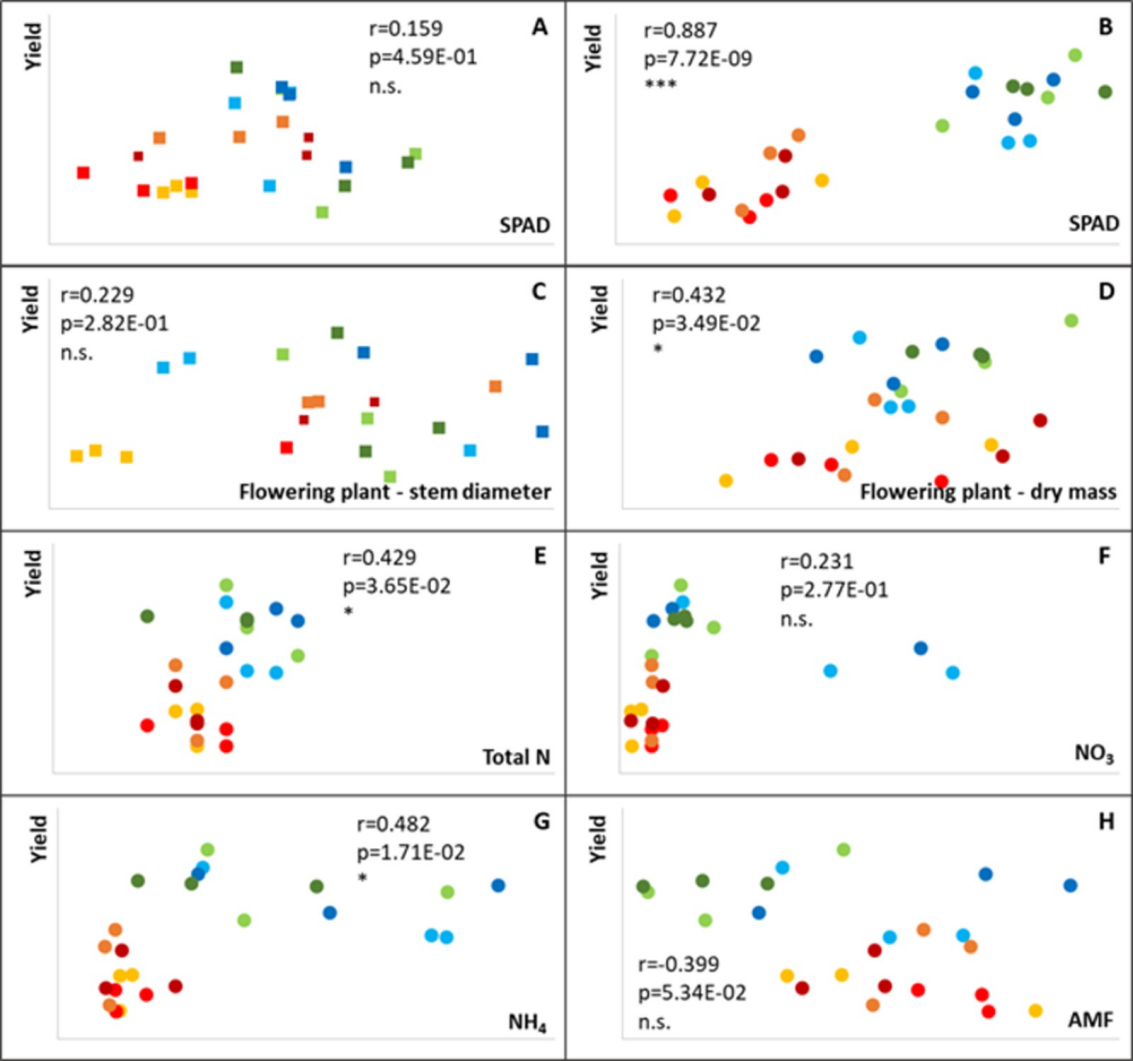
Pearson’s product-moment correlation analysis between crop yield and N forms in the soil (E-G) and between crop yield and early plant growth parameters (C, D), SPAD (A, B) values or AMF colonization, M% (H), for maize (A, C) and wheat (B, D, E, F, G, H). The key of the symbols is the same as at Fig 4. (Control–red, N–blue, P–yellow, NPK–green, light colours: Without FM manure, dark colours: With FM manure). n.s.: non-significant, *: p<0.05, **: p<0.01, ***: p<0.001.

A relationship was detected between the soil N and N forms and the wheat grain yield (Fig 7E and 7G), but this was not as clear as the correlation between the chlorophyll content of the flowering plant and the grain yield of the harvested crop. Chlorophyll content proved to be a more effective predictor of grain yield than any other plant growth parameters (Fig 7B and 7D).

We observed some differences in the AMF root colonization between the two years, as is normal in the case of different crops. We found that the AMF dependence was higher for maize (mean M value: 34%) than for wheat (M: 24%). Seasonal dynamics can also be seen for annual plants: the maximum values typically being measured in the roots of flowering plants. A treatment effect was found for complex fertilizers (farmyard manure and NPK). The negative effect of NPK fertilizer was visible (Fig 7H) and significant (Fig 2), while the effect of farmyard manure was detected at harvest in 2018 (Fig 3).

## Discussion

Agricultural soils are exposed to recurrent disturbances during intensive management, including tillage, crop cultivation and harvest, plant protection, and fertilization. These processes may impact the functional diversity and abundance of microbial communities [44–47].

The most evident modifications in soil characteristics related to fertilization are changes in the soil nutrients levels. In addition, unbalanced or excess fertilization may alter the soil pH and electrical conductivity. This work presents that tendentious differences in soil properties after farmyard manure and P fertilizer treatments, while we detected more pronounced shifts after N fertilization treatment (Fig 2). Besides a higher amount of total N, ammonium-N, and nitrate-N in the soil, other well-documented soil processes, such as acidification and a decrease in cation exchange capacity, may appear as the result of mineral N addition. Especially when the N fertilizer efficiency is low, a considerable increase in acidity may be generated by nitrification [48–51]. The PCA analysis demonstrated the main effect of soil pH (Fig 4), which was correlated with soil N forms or exchangeable cations (Ca^2+^) and determined the direction of the citrate utilization vector and the evenness factor calculated from the SIR values. The citrate utilization capacity of rhizosphere microorganisms showed a close correlation with soil pH, the same as it was already stated in other experiment results on citric acid [52–54], and on other acidic substrates, such as malic acid or ascorbic acid [55]. The changes in these soil parameters were closely correlated with each other, whereas no factors acting on biological parameters and no cause-and-effect relationships were revealed by PCA analysis (Fig 4). Correlation analysis was therefore performed for soil parameters in question and for the citrate utilization rate. We found the most significant correlation between citrate utilization and soil pH (Fig 6). An increase in N (NH_4_^+^) fertilization generally induces pH reduction, which may have contributed to a change in bacterial communities [28]. For example a higher relative abundance of acidotolerant taxa [56] with a possibly modified citrate utilization pattern. Increased N fertilization may also result in a P-limited rhizosphere, so citrate addition may have contributed to accelerated soil metabolism via phosphate mobilization, by enhancing the dissolution and desorption of phosphate (and possibly other ions) [57–59]. Changes in chemical soil parameters may alter soil microbial communities and functional diversity as well, however functional diversity may shift without any changes in the taxonomic composition [60,61].

Functional evenness (E) is usually reduced by land use, soil tillage, and especially by mineral fertilization [52,55]. In the present experiment, the pronounced effect of soil pH on the citrate utilization rate decreased the functional evenness index, since citrate produced an outlier in the substrate utilization data set and generated unevenness between the substrates. Besides the change in citrate utilization, other carboxylic acids, e.g,. malic acid or succinate, also modified the E factor in N-fertilized treatments. Wahbi et al. [62] also found that carboxylic acid substrate utilization rates were the most sensitive to treatments in agricultural soils and suggested that this was due to the effect of root exudates and AMF colonization changes. However, this was not confirmed by the present results, as the different crops with diverse AMF dependence in different years produced similar SIR patterns for citrate utilization, and AMF colonization exhibited no correlation with the SIR values of carboxylic acids. We only detected AMF colonization changes in the case of balanced plant nutrition treatments. The most pronounced decrease was measured in the roots of flowering maize plants treated with NPK fertilizers and farmyard manure, where the root colonization (M%) was only 10% in the treated plants, while unbalanced or no fertilization resulted in 30–40% colonization rates. As previously described in several studies [24,63–65], this implies a plastic plant-mediated effect on AMF colonization, depending on plant requirements.

By causing shifts in the substrate utilization pattern [52,55] or increasing the species diversity, characterized by DGGE profiles [8], organic fertilizers can have an impact on the taxonomic and functional diversity of soil microbiota. In the present work, the first sampling was done eighteen months after the last manure addition, so only the long-term effects of farmyard manure could be analysed. In contrast to chemical soil parameters, and the impact of chemical fertilization (Figs 2 and 4), the cumulative effect of repeated manuring on soil microbiota and biological parameters seems to be marginal, although it cannot be ruled out, that the organic manure has short-term effects as well.

Among the examined substrates only citrate utilization showed a clear treatment effect, however, other tendencies were also observed. Both SIR and the utilization rates of amino acids and sugars changed seasonally. Neutral sugar utilization is usually correlates with microbial biomass [66], and a higher rate of sugar utilization implies a higher abundance of r-strategist microbes [67]. The higher total SIR during flowering and at harvest, therefore, suggested higher microbial biomass, which may have been the consequence of a higher amount of root exudates or plant residues in the soil during this period. Despite the higher total SIR values at these sampling times, the utilization rate of amino acids was relatively low compared to that of sugars (Fig 4). Apart from the effect of citrate, differences in the utilization rates of amino acids and sugars (PC2) explained most of the variation in the PCA analysis of the 23 substrates at flowering for wheat (Fig 5). The higher rate of amino acid utilization, especially asparagine, was more characteristic of NPK than N-treated plots.

N fertilization is fundamental for crop yield, therefore, significantly higher crop production is normally expected on N- or NPK-treated plots [1]. However, the extra yield was only detected in the second year, because yield stability was influenced by several other factors, e.g., meteorological conditions and the effects of previous crops. Treatment effects were reduced by water deficit during plant growth and by atmospheric drought in 2017. The treatment effect on wheat is expected to be higher when maize is the previous crop, as maize requires a larger soil N pool, and the mineralization period after maize is shorter due to the later harvesting time [1].

Nitrogen supply and plant chlorophyll contents are closely related parameters [68,69]. The SPAD value measured on plant leaves in the early growth stage can be a useful parameter for yield prediction [70]. Indeed, the SPAD values in the flowering stage showed a closer correlation with wheat yield than soil or plant growth parameters (Fig 7). This correlation was not significant for maize in 2017, as no effect of N fertilization on yield could be detected that year.

## Conclusion

We detected some characteristic changes in biological parameters in the long-term fertilization experiment. Although no sensitive, detailed fingerprint of CLPP was demonstrated using MicroResp^TM^ in this case, selected parameters from CLPP (e.g., citrate utilization, total SIR value or the ratio of utilization of amino acids and sugars) responded to fertilizer treatments or seasonal changes. Suggesting a plant-mediated effect of mycorrhiza, AMF colonization seemed to be dependent on plant nutrition status and requirements, however the soil acidification induced by mineral N fertilization proved to be the main factor responsible for changes in the catabolic activity patterns of soil microbiota.

Our study suggests that soil acidification is not only a main cause of agricultural soil degradation, but also the greatest adversary of soil microbial resilience. This fact further emphasizes the importance of avoiding excess ammonia in the soil during mineral fertilization.

## Supporting information

S1 Fig Calibration curve with the relationship of measured CO_2_ and absorbance of indicator plate. The Harris-model (pink) and the modified Harris-model (red). The used calibration curve is the combination of empty and full red dots. The photo in background is an indicator plate with the sign of added substrates.
(TIF)

S1 File Tables and figures of data and tables of statistical analysis. Tables contain the basic data of measured parameters, figures show the mean values of measured parameters and added tables contain the data of statistical analysis (F1-F4: Factors of the ANOVA. Factor 1 (F1): Comparison of the 2 years. Factor 2 (F2): Comparison of sampling times in the season. Factor 3 (F3): Comparison of treatments with and without farmyard manure. Factor (F4): Comparison of mineral fertilizers treatments. M: Maize, W: Wheat, E: Early sampling, F: Flowering time, H: Harvest, FM: Farmyard manure, FMØ: Without farmyard manure, C: Control (no mineral fertilizer), N: Nitrogen fertilizer, P: Phosphorus fertilizer, NPK: Nitrogen, phosphorus and potassium fertilizer).
(DOCX)

## References

[1] Árendás T, Bóni P, Csathó P, Molnár D, Berzsenyi Z. Fertiliser responses of maize and winter wheat as a function of year and forecrop. Acta Agron Hung. 2010; 58: 109–114. 10.1556/AAgr.58.2010.Suppl.1.16.

[2] Berzsenyi Z, Győrffy B, Lap D. Effect of crop rotation and fertilisation on maize and wheat yields and yield stability in a long-term experiment. Eur J Agron. 2000; 13(2–3): 225–244. 10.1016/S1161-0301(00)00076-9

[3] Micskei G, Jocsak I, Arendas T, Bonis P, Berzsenyi Z. Effect of farmyard manure and mineral fertiliser on the yield and yield components of maize in a long-term monoculture experiment in Martonvásár. Acta Agron Hung. 2010; 58(Supplement 1): 63–68. 10.1556/AAgr.58.2010.Suppl.1.9.

[4] Körschens M, Albert E, Armbruster M, Barkusky D, Baumecker M, Behle-Schalk L et al. Effect of mineral and organic fertilization on crop yield, nitrogen uptake, carbon and nitrogen balances, as well as soil organic carbon content and dynamics: results from 20 European long-term field experiments of the twenty-first century. Arch Agron Soil Sci. 2013; 59(8): 1017–1040. 10.1080/03650340.2012.704548.

[5] Bai Z, Caspari T, Gonzalez MR, Batjes NH, Mäder P, Bünemann EK et al. Tóth Z. Effects of agricultural management practices on soil quality: A review of long-term experiments for Europe and China. Agr Ecosyst Environ. 2018; 265: 1–7. 10.1016/j.agee.2018.05.028.

[6] Diacono M, Montemurro F. Long-Term Effects of Organic Amendments on Soil Fertility, in: Lichtfouse E, Hamelin M, Navarrete M, Debaeke P (Eds) Sustainable Agriculture. 2011; Volume 2. Springer, Dordrecht, pp. 761–786. 10.1007/978-94-007-0394-0_34.

[7] Wang Y, Wang E, Wang D, Huang S, Ma Y, Smith CJ, Wang L. Crop productivity and nutrient use efficiency as affected by long-term fertilisation in North China Plain. Nutr Cyc Agroecosys. 2010; 86(1): 105–119. 10.1007/s10705-009-9276-5.

[8] Gu Y, Zhang X, Tu S, Lindström K (2009) Soil microbial biomass, crop yields, and bacterial community structure as affected by long-term fertilizer treatments under wheat-rice cropping. Eur J Soil Biol. 2009; 45(3): 239–246. 10.1016/j.ejsobi.2009.02.005.

[9] Hamel C, Hanson K, Selles F, Cruz AF, Lemke R, McConkey B, Zentner R. Seasonal and long-term resource-related variations in soil microbial communities in wheat-based rotations of the Canadian prairie. Soil Biol Biochem. 2006; 38(8): 2104–2116. 10.1016/j.soilbio.2006.01.011.

[10] van der Bom F, Nunes I, Raymond NS, Hansen V, Bonnichsen L, Magid J et al. Long-term fertilization form, level and duration affect the diversity, structure and functioning of soil microbial communities in the field. Soil Biol Biochem. 2018; 122: 91–103. 10.1016/j.soilbio.2018.04.003.

[11] Allison SD, Martiny JB. Resistance, resilience, and redundancy in microbial communities. P Nati A Sci. 2008; 105(Supplement 1) 11512–11519. 10.1073/pnas.0801925105. PMC2556421 18695234

[12] Chu H, Lin X, Fujii T, Morimoto S, Yagi K, Hu J, Zhang J. Soil microbial biomass, dehydrogenase activity, bacterial community structure in response to long-term fertilizer management. Soil Biol Biochem. 2007; 39(11): 2971–2976. 10.1016/j.soilbio.2007.05.031.

[13] Zhao J, Ni T, Li Y, Xiong W, Ran W, Shen B, Shen Q, Zhang R. Responses of bacterial communities in arable soils in a rice-wheat cropping system to different fertilizer regimes and sampling times. PLoS ONE. 2014; 9(1): e85301. doi: 10.1371/journal.pone.0085301 24465530 PMC3896389

[14] Hartmann M, Fliessbach A, Oberholzer HR, Widmer F. Ranking the magnitude of crop and farming system effects on soil microbial biomass and genetic structure of bacterial communities. FEMS Microbiol Ecol. 2006; 57(3): 378–388. doi: 10.1111/j.1574-6941.2006.00132.x 16907752

[15] Girvan MS, Bullimore J, Ball AS, Pretty JN, Osborn AM. Responses of active bacterial and fungal communities in soils under winter wheat to different fertilizer and pesticide regimens. Appl Environ Microb. 2004; 70(5): 2692–2701. doi: 10.1128/AEM.70.5.2692-2701.2004 15128520 PMC404392

[16] Zhang L, Xu Z. Assessing bacterial diversity in soil. J Soil Sediment. 2008; 8(6): 379–388. 10.1007/s11368-008-0043-z.

[17] Ros M, Klammer S, Knapp B, Aichberger K, Insam H. Long‐term effects of compost amendment of soil on functional and structural diversity and microbial activity. Soil Use Manage. 2006. 22(2): 209–218. 10.1111/j.1475-2743.2006.00027.x.

[18] Koorem K, Gazol A, Öpik M, Moora M, Saks Ü, Uibopuu A, et al. Soil Nutrient Content Influences the Abundance of Soil Microbes but Not Plant Biomass at the Small-Scale. PLoS ONE. 2014; 9(3): e91998. doi: 10.1371/journal.pone.0091998 24637633 PMC3956881

[19] Fadiji AE, Babalola OO. Metagenomics methods for the study of plant-associated microbial communities: a review. J Microbiol Meth. 2020; 170: 105860. 10.1016/j.mimet.2020.105860. 32027927

[20] Jones DL, Hill PW, Smith AR, Farrell M, Ge T, Banning NC, Murphy DV. Role of substrate supply on microbial carbon use efficiency and its role in interpreting soil microbial community-level physiological profiles (CLPP). Soil Biol Biochem. 2018; 123: 1–6. 10.1016/j.soilbio.2018.04.014.

[21] Chapman SJ, Campbell CD, Artz RR. Assessing CLPPs using MicroResp™. J Soils Sediments. 2007; 7: 406–410. 10.1065/jss2007.10.259.

[22] Brundrett M, Tedersoo L. Misdiagnosis of mycorrhizas and inappropriate recycling of data can lead to false conclusions. New Phytol. 2019; 221(1): 18–24. doi: 10.1111/nph.15440 30191568

[23] Giovannetti M, Sbrana C. Meeting a non-host: the behaviour of AM fungi. Mycorrhiza. 1998; 8(3): 123–130. 10.1007/s005720050224.

[24] Füzy A, Tóth T, Biró B. Mycorrhizal colonisation can be altered by the direct and indirect effect of drought and salt in a split root experiment. Cereal Res Commun. 2007; 35(2): 401–404. 10.1556/CRC.35.2007.2.59.

[25] Püschel D, Bitterlich M, Rydlová J, Jansa J. Drought accentuates the role of mycorrhiza in phosphorus uptake. Soil Biol Biochem. 2021; p108243. 10.1016/j.soilbio.2021.108243.

[26] van der Hiejden MGA, Bardgett RD, van Straalen NM. The unseen majority: soil microbes as drivers of plant diversity and productivity in terrestrial ecosystems. Ecol Lett. 2008; 11: 29. 10.1111/j.1461-0248.2007.01139.x. 18047587

[27] Gosling P, Mead A, Proctor M, Hammond JP, Bending GD. Contrasting arbuscular mycorrhizal communities colonizing different host plants show a similar response to a soil phosphorus concentration gradient. New Phytol. 2013; 198(2): 546–556. doi: 10.1111/nph.12169 23421495 PMC3798118

[28] Wei X, Zhu Z, Liu Y, Luo Y, Deng Y, Xu X et al. C: N: P stoichiometry regulates soil organic carbon mineralization and concomitant shifts in microbial community composition in paddy soil. Biol. Fert Soils. 2020; 56(8): 10.1007/s00374-020-01468-7.

[29] Li L, Xu M, Eyakub Ali M, Zhang W, Duan Y, Li D. Factors affecting soil microbial biomass and functional diversity with the application of organic amendments in three contrasting cropland soils during a field experiment. PLoS ONE. 2018; 13(9): e0203812. doi: 10.1371/journal.pone.0203812 30212559 PMC6136761

[30] IUSS Working Group. World Reference Base (WRB) for Soil Resources. International soil classification system for naming soils and creating legends for soil maps. World Soil Resources Reports. 2015; 106 FAO Rome.

[31] Gy Füleky, Rajkai-Végh K. Nutrient providing capacity of soil. in: Gy Füleky (ed) Nutrient management. Mezőgazda Kiadó, Budapest. 1999; 91–139.

[32] Meier U. BBCH-Monograph: growth stages of mono-and dicotyledonous plants (p 158). Technical Report 2 Edn. Federal Biological Research Centre for Agriculture and Forestry. 2001.

[33] MSZ-08-0210-2:1977. Testing organic carbon content in soils Hungarian Standard Institution: Budapest 1977.

[34] MSZ-08-0206/2:1978. Evaluation of Some Chemical Properties of the Soil: Laboratory Tests (Ph Value, Phenolphtalein Alkalinity Expressed in Soda, Total Water Soluble Salt Content, Hydrolytic (Y1 Value) and Exchangeable Acidity (Y2 Value). Hungarian Standard Institution: Budapest 1978.

[35] MSZ-20135:1999. Determination of the soluble nutrient element content of the soil. Hungarian Standard Institution: Budapest 1999.

[36] Egner H, Riehm H, Domingo W. Investigations on the chemical soil analysis as a basis for assessing the soil nutrient status. II: Chemical extraction methods for phosphorus and potassium determination. Kungliga Lantbrukshügskolans Annaler. 1960; 26: 199–215.

[37] ISO-11261 Soil quality-Determination of total nitrogen—Modified Kjeldahl method. Geneva, Switzerland, 1995; ISO 4.

[38] Campbell CD, Chapman SJ, Cameron CM, Davidson MS, Potts JM. A rapid microtiter plate method to measure carbon dioxide evolved from carbon substrate amendments so as to determine the physiological profiles of soil microbial communities by using whole soil. Appl Environ Microb. 2003; 69(6): 3593–3599. 10.1128/AEM.69.6.3593-3599.2003. PMC161481 12788767

[39] Magurran AE. Ecological Diversity and its Measurement. Princeton university press. 1988; ISBN: 978-94-015-7358-0.

[40] Phillips JM, Hayman DS. Improved procedures for clearing roots and staining parasitic and VAM fungi for rapid assessment of infection. T Brit Mycol Soc. 1970; 55: 158–161. 10.1016/s0007-1536(70)80110-3.

[41] Trouvelot A, Kough JL, Gianinazzi-Pearson V. Mesure du taux de mycorhization VA d’un systeme radiculaire. Recherche de methodes d’estimation ayant une significantion fonctionnelle. in: Gianinazzi-Pearson V, Gianinazzi S (Eds) Physiological and genetic aspects of mycorrhizae. 1986; pp. 217–221. INRA Paris.

[42] R Core Team. R: A language and environment for statistical computing. R Foundation for Statistical Computing, Vienna Austria. 2019; URL https://www.R-project.org/.

[43] Wobbrock JO, Findlater L, Gergle D, Higgins JJ. The aligned rank transform for nonparametric factorial analyses using only anova procedures. In Proceedings of the SIGCHI conference on human factors in computing systems. 2011; 143–146. 10.1145/1978942.1978963.

[44] Bolo P, Kihara J, Mucheru-Muna M, Njeru EM, Kinyua M, Sommer R. Application of residue, inorganic fertilizer and lime affect phosphorus solubilizing microorganisms and microbial biomass under different tillage and cropping systems in a Ferralsol. Geoderma. 2021; 390: 114962. 10.1016/j.geoderma.2021.114962.

[45] Chakraborty A, Chakrabarti K, Chakraborty A, Ghosh S. Effect of long-term fertilizers and manure application on microbial biomass and microbial activity of a tropical agricultural soil. Biol Fertil Soils. 2011; 47(2): 227–233. 10.1007/s00374-010-0509-1.

[46] Gazdag O, Kovács R, Parádi I, Füzy A, Ködöböcz L, Mucsi M et al. Density and diversity of microbial symbionts under organic and conventional agricultural management. Microbes Environ. 2019; ME18138. doi: 10.1264/jsme2.ME18138 31189767 PMC6759338

[47] Gong W, Yan X, Wang J, Hu T, Gong Y. Long-term manure and fertilizer effects on soil organic matter fractions and microbes under a wheat–maize cropping system in northern China. Geoderma. 2009; 149(3–4): 318–324. 10.1016/j.geoderma.2008.12.010.

[48] Barak P, Jobe BO, Krueger AR, Peterson LA, Laird DA (1997) Effects of long-term soil acidification due to nitrogen fertilizer inputs in Wisconsin. Plant Soil. 1997; 197(1): 61–69. 10.1023/A:1004297607070.

[49] Waldrop MP, Zak DR, Sinsabaugh RL. Microbial community response to nitrogen deposition in northern forest ecosystems. Soil Biol Biochem. 2004; 36:1443–1451. 10.1016/j.soilbio.2004.04.023.

[50] Chen DM, Lan ZC, Hu SJ, Bai YF. Effects of nitrogen enrichment on belowground communities in grassland: relative role of soil nitrogen availability vs. soil acidification. Soil Biol Biochem. 2015; 89: 99–108. 10.1016/j.soilbio.2015.06.028.

[51] Kim DG, Saggar S, Roudier P. The effect of nitrification inhibitors on soil ammonia emissions in nitrogen managed soils: a meta-analysis. Nutr Cyc Agroecosys. 2012; 93(1): 51–64. 10.1007/s10705-012-9498-9.

[52] Sradnick A, Murugan R, Oltmanns M, Raupp J, Joergensen RG. Changes in functional diversity of the soil microbial community in a heterogeneous sandy soil after long-term fertilization with cattle manure and mineral fertilizer. Appl Soil Ecol. 213; 63: 23–28. 10.1016/j.apsoil.2012.09.011.

[53] Zhou X, Wu H, Koetz E, Xu Z, Chen C. Soil labile carbon and nitrogen pools and microbial metabolic diversity under winter crops in an arid environment. Appl Soil Ecol. 2012; 53: 49–55. 10.1016/j.apsoil.2011.11.002.

[54] Creamer RE, Stone D, Berry P, Kuiper I. Measuring respiration profiles of soil microbial communities across Europe using MicroResp™ method. Appl Soil Ecol. 2016; 97: 36–43. 10.1016/j.apsoil.2015.08.004.

[55] Romaniuk R, Giuffré L, Costantini A, Nannipieri P. Assessment of soil microbial diversity measurements as indicators of soil functioning in organic and conventional horticulture systems. Ecol Indic. 2011; 11(5): 1345–1353. 10.1016/j.ecolind.2011.02.008.

[56] Megyes M, Borsodi AK, Árendás T, Márialigeti K. Variations in the diversity of soil bacterial and archaeal communities in response to different long-term fertilization regimes in maize fields. Appl Soil Ecol. 2021; 168: 104120. 10.1016/j.apsoil.2021.104120.

[57] Chen T, Philips C, Hamilton J, Chartbrand B, Grosskleg J, Bradshaw K et al. Citrate addition increased phosphorus bioavailability and enhanced gasoline bioremediation. J Environ Qual. 2017; 46(5): 975–983. doi: 10.2134/jeq2017.02.0064 28991988

[58] Shen F, Wu J, Fan H, Liu W, Guo X, Duan H et al. Soil N/P and C/P ratio regulate the responses of soil microbial community composition and enzyme activities in a long-term nitrogen loaded Chinese fir forest. Plant Soil. 2019; 436(1): 91–107. 10.1007/s11104-018-03912-y.

[59] Wei L, Chen C, Xu Z. Citric acid enhances the mobilization of organic phosphorus in subtropical and tropical forest soils. Biol. Fertil Soils. 2010; 46(7): 765–769. 10.1007/s00374-010-0464-x.

[60] Wertz S, Degrange V, Prosser JI, Poly F, Commeaux C, Freitag T et al. Maintenance of soil functioning following erosion of microbial diversity. Environ. Microbiol. 2006; 8(12): 2162–2169. doi: 10.1111/j.1462-2920.2006.01098.x 17107557

[61] Nielsen UN, Ayres E, Wall DH, Bardgett RD. Soil biodiversity and carbon cycling: a review and synthesis of studies examining diversity–function relationships. Europ J Soil Sci. 2011; 62(1): 105–116. 10.1111/j.1365-2389.2010.01314.x.

[62] Wahbi S, Prin Y, Thioulouse J, Sanguin H, Baudoin E, Maghraoui T et al. Impact of wheat/faba bean mixed cropping or rotation systems on soil microbial functionalities. Front Plant Sci. 2016; 7: 1364. doi: 10.3389/fpls.2016.01364 27695462 PMC5023684

[63] Bhadalung NN, Suwanarit A, Dell B, Nopamornbodi O, Thamchaipenet A, Rungchuang J. (2005) Effects of long-term NP-fertilization on abundance and diversity of arbuscular mycorrhizal fungi under a maize cropping system. Plant Soil. 2005; 270(1): 371–382. 10.1007/s11104-004-1829-4.

[64] Füzy A, Biró B, Tóth T, Hildebrandt U, Bothe H. Drought, but not salinity, determines the apparent effectiveness of halophytes colonized by arbuscular mycorrhizal fungi. J Plant Physiol. 2008; 165(11): 1181–1192. doi: 10.1016/j.jplph.2007.08.010 18155803

[65] Treseder KK, Allen MF. Direct nitrogen and phosphorus limitation of arbuscular mycorrhizal fungi: a model and field test. New Phytol. 2002; 155(3): 507–515. doi: 10.1046/j.1469-8137.2002.00470.x 33873310

[66] Lin Q, Brookes PC. Comparison of substrate induced respiration, selective inhibition and biovolume measurements of microbial biomass and its community structure in unamended, ryegrass-amended, fumigated and pesticide-treated soils. Soil Biol Biochem. 1999; 31(14). 10.1016/S0038-0717(99)00122-4.

[67] Degens BP, Schipper LA, Sparling GP, Vojvodic-Vukovic M. Decreases in organic C reserves in soils can reduce the catabolic diversity of soil microbial communities. Soil Biol Biochem. 2000; 32(2): 189–196. 10.1016/S0038-0717(99)00141-8.

[68] Hawkins JA, Sawyer JE, Barker DW, Lundvall JP. Using relative chlorophyll meter values to determine nitrogen application rates for corn. Agron J. 2007; 99(4): 1034–1040. 10.2134/agronj2006.0309.

[69] Houles V, Guerif M, Mary B. Elaboration of a nitrogen nutrition indicator for winter wheat based on leaf area index and chlorophyll content for making nitrogen recommendations. Eur J Agron. 2007; 27(1): 1–11. 10.1016/j.eja.2006.10.001.

[70] Monostori I, Árendás T, Hoffman B, Galiba G, Gierczik K, Szira F et al. Relationship between SPAD value and grain yield can be affected by cultivar, environment and soil nitrogen content in wheat. Euphytica. 2016; 211(1): 103–112. 10.1007/s10681-016-1741-z.

