# Supplementary figures and images for: Soil biological activity after a sixty-year fertilization practice in a wheat-maize crop rotation
Source: PLoS One. 2023 Sep 28;18(9):e0292125. doi: 10.1371/journal.pone.0292125 (PMC10538786; doi:10.1371/journal.pone.0292125)

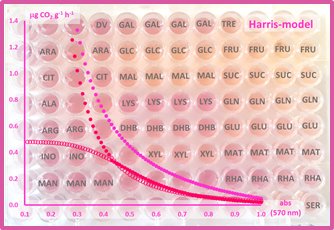

Supplement: S1 Fig — The Harris-model (pink) and the modified Harris-model (red). The used calibration curve is the combination of empty and full red dots. The photo in background is an indicator plate with the sign of added substrates. (TIF) [file pone.0292125.s001.tif]
