# Supplementary material for: Soil biological activity after a sixty-year fertilization practice in a wheat-maize crop rotation
Source: PLoS One. 2023 Sep 28;18(9):e0292125. doi: 10.1371/journal.pone.0292125 (PMC10538786; doi:10.1371/journal.pone.0292125)
Supplement: S1 File — Tables contain the basic data of measured parameters, figures show the mean values of measured parameters and added tables contain the data of statistical analysis (F1-F4: Factors of the ANOVA. Factor 1 (F1): Comparison of the 2 years. Factor 2 (F2): Comparison of sampling times in the season. Factor 3 (F3): Comparison of treatments with and without farmyard manure. Factor (F4): Comparison of mineral fertilizers treatments. M: Maize, W: Wheat, E: Early sampling, F: Flowering time, H: Harvest, FM: Farmyard manure, FMØ: Without farmyard manure, C: Control (no mineral fertilizer), N: Nitrogen fertilizer, P: Phosphorus fertilizer, NPK: Nitrogen, phosphorus and potassium fertilizer). (DOCX) [file pone.0292125.s002.docx]

**S1 Data tables and figures.**

Soil parameters

Plant physiology and plant growth

AMF symbiosis

Community Level Physiological Profiles

*Sugars*

*Amino acids*

*Poliols*

*Carboxyl acids*
